# How a target’s speed influences the extent to which the time or place at which it is intercepted is adjusted

**DOI:** 10.1007/s00221-025-07108-6

**Published:** 2025-06-09

**Authors:** Giorgia Bertonati, Monica Gori, Jeroen B.J. Smeets, Eli Brenner

**Affiliations:** 1 Unit for Visually Impaired People (UVIP), Istituto Italiano di Tecnologia, Genoa, Italy; 2 Department of Human Movement Sciences, Vrije Universiteit Amsterdam, Amsterdam, The Netherlands; 3 DIBRIS Department, Università degli studi di Genova, Genoa, Italy

**Keywords:** Interception, Feedback, Arm movements, Velocity, Position, Timing

## Abstract

Goal-directed movements are constantly guided by the latest information about the target’s position. Nevertheless, movements seldom end perfectly on target, so subsequent movements are adjusted to avoid repeating errors. One could intercept moving targets at different positions at different times, so one could adjust both the position and the timing of the endpoint of both the current and the next movement. It could be advantageous to rely more on adjusting the timing for faster targets, because for faster targets a change in timing corresponds with a larger change in position. We therefore examined how participants responded to ‘errors’ that were introduced by having slow and fast targets jump slightly backwards or forwards along their path. If there was enough time to adjust the ongoing movement after the jump, timing was indeed responsible for a larger fraction of the adjustment for fast targets. But the actual change in timing did not depend on the target’s speed. The same change in timing compensated for a larger part of the error for fast targets, so the position could change less. If there was not enough time to adjust the ongoing movement, neither the timing nor the position on the next trial changed differently for the different target speeds. Consequently, a larger fraction of the error was compensated for if the target moved faster. Thus, how people adjust their timing does not depend on the target’s speed, but the same change to the timing has more impact if the target is moving faster.

## Introduction

It is well established that goal-directed arm movements are more precise if the target and hand are visible (Carlton 1981; Dessing et al. 2009; Elliott et al. 1991, 1994; López-Moliner et al. 2010; Ma-Wyatt and McKee 2007; Prablanc et al. 1979; Whiting and Sharp 1974; Woodworth 1899). This is not surprising, because people constantly adjust their ongoing movements to the latest visually perceived position of the target (Brenner et al. 2023; Goodale et al. 1986; Prablanc and Martin 1992) and of their hand (Brenner and Smeets 2023; Cámara et al. 2018; Saunders and Knill 2005). Besides using visual information to guide the ongoing movement to the target, people also use visually perceived endpoint errors to adjust subsequent movements (Brenner et al. 2023; Redding and Wallace 2003; van Beers 2009; van den Dobbelsteen et al. 2003; van der Kooij et al. 2013). If the target of the movement is moving, one can select from many possible combinations of when and where to reach it. If new visual information reveals that a target has moved further than anticipated, people adjust their movement to intercept the target further along its path, or earlier, or some combination of both (Brenner and Smeets 2015). If there is not enough time to adjust the ongoing movement, people aim further along the next target’s path, intercept the next target earlier, or some combination of both (Brenner et al. 2023). Does the way in which temporal and spatial adjustments are combined matter?

**Fig. 1:**
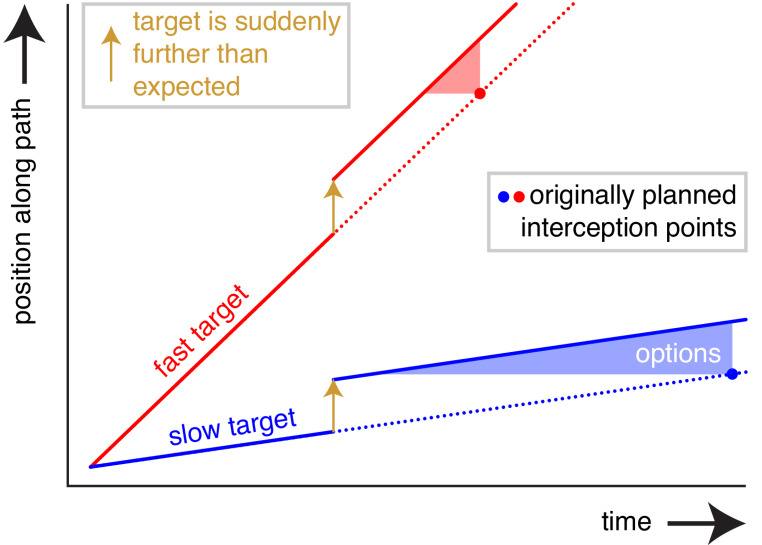
Why one might better adjust *when* one reaches fast targets and *where* one reaches slow targets. Consider a target that is suddenly perceived to be further than expected for some reason (vertical arrows; in the experiments this will be because we make the target suddenly jump). To successfully intercept the target, one must compensate for the discrepancy between its originally anticipated trajectory (dotted lines) and the trajectory based on the newly perceived position (solid lines). One has many options for doing so, because one can intercept the target at any point along the solid line, so with various combinations of adjustments to position and time. The shapes of the shaded triangles reveal that any change in position (vertical size) corresponds with a larger change in time (horizontal size) if the target is moving more slowly (blue rather than red triangle)

We suggest that how one combines temporal and spatial adjustments might matter, because it is advantageous for adjustments to be small (Liu and Todorov 2007). Consider that you planned to intercept a moving target at a certain point, but you suddenly see that the target is further than you expected (Fig. 1). If the target is moving very slowly, it makes sense to mainly change where you try to reach it, because otherwise you would have to change the timing a lot to adjust the movement to the new information about the target’s position. If the target is moving fast, it might be better to change when you try to reach the target, because a small change in timing could already adjust the movement enough to account for the new target information. This reasoning does not only hold for adjusting the ongoing movement, but also for learning to move differently on subsequent trials. One might therefore expect people to primarily adjust the position for slow targets and the timing for fast targets, both when adjusting an ongoing movement and when learning how to deal with the next movement. Do they?

We previously found that people could adjust both when and where they tried to tap on moving targets when confronted with small jumps in the target’s position (Brenner and Smeets 2015). We also previously found that people could adjust both when and where they try to hit the next target when confronted with errors that they made by ignoring the fact that the target was accelerating (Brenner et al. 2016, 2023). There are ample other examples of people learning to aim at a different position (e.g. Shadmehr et al. 2010; Tseng et al. 2007; van Beers 2009; van der Kooij et al. 2015) or to time the movement differently (e.g. Brenner and Smeets 2011; Langley and Zelaznik 1984) on the basis of feedback. Thus, people can change the time and the place at which they try to hit moving targets, both when guiding an ongoing movement and when adjusting the next movement. Here, we examine whether the target’s speed influences the balance between adjusting when and where. Do people adjust the timing more for faster targets? Is the balance the same for guiding an ongoing movement as for adjusting the next movement?

## Materials and methods

**Fig. 2:**
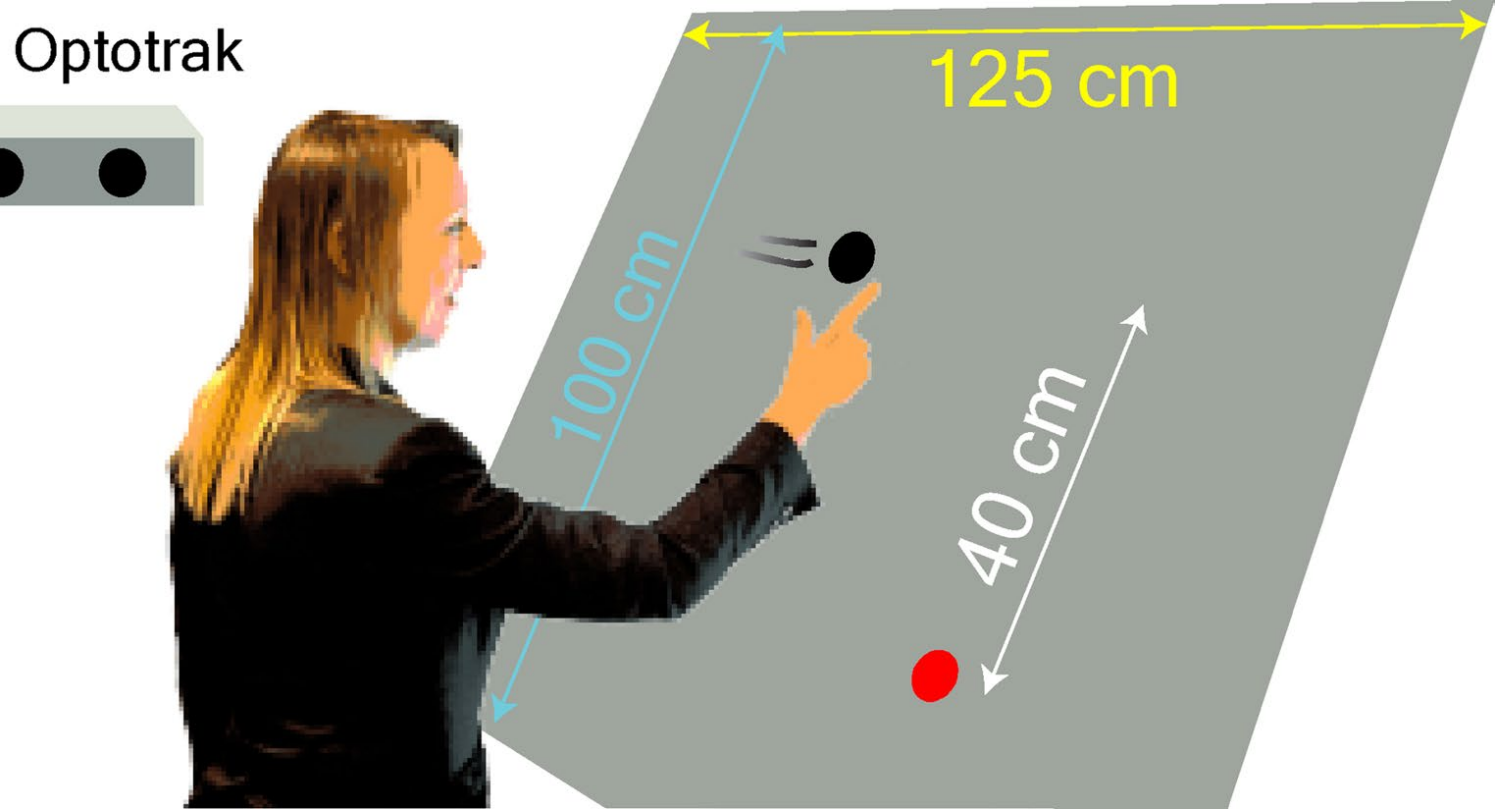
Schematic representation of a participant in Experiment 1. Each trial started with the participant placing his or her right index finger at the starting point (red disk). Briefly after that, a black, moving target disk appeared. The task was to tap on the target disk

Participants stood in front of a large screen and tried to intercept moving targets by lifting their right index finger off an indicated starting point, 20 cm below the centre of the screen, and tapping on the target. The target moved to the right at a constant velocity, 40 cm above the starting point (Fig. 2). Participants had to wait for the moving target to appear before lifting their finger. To interpret participants’ adjustments to their ongoing or subsequent movements, we have to know how much they should have adjusted. We therefore artificially introduced a need to adjust the movement by having the moving target jump along its path.

There were two experiments, that mainly differed in the timing of the jump. In Experiment 1, the jump was initiated as soon as the participant’s finger left the outline of the starting point, so there was ample time to adjust the ongoing movement (Brenner et al. 2023). In Experiment 2, the jump was initiated when the participant’s finger was within 5 cm of the target’s path. Considering a delay of between 22 and 60 ms from the moment the requirements were met to an actual change occurring on the screen (Brenner and Smeets 2022), the target jump was only visible on the screen near the moment the finger hit the screen. Thus, participants could not adjust their ongoing movement. But they could use the feedback to adjust the next trial. Experiment 2 included a control in which participants were encouraged to change the moment rather than the position at which they intercepted the target by indicating about where the target had to be hit.

### Participants

There were 44 participants in Experiment 1, 24 in the main part of Experiment 2, and 17 in the control of Experiment 2. Most were between 18 and 30 years old, but a few were older (up to 65 years old). There were slightly more females than males (about 60%). They all had normal or corrected-to-normal vision, and none had evident motor abnormalities.

### Stimuli and experiments

**Fig. 3:**
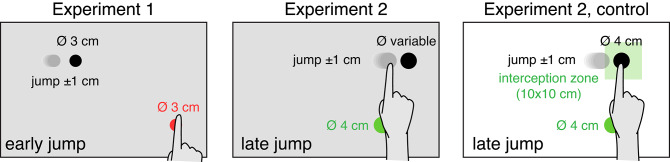
Schematic representation of the experiments (not to scale) showing the colours and diameters (Ø) of the starting points and targets. The target and finger are shown at the moment that the target jumped (jumps to the right are shown, but the target could also jump to the left). The grey disks represent earlier target positions. In Experiment 1 the target jumped as soon as the finger started moving, so there was enough time to adjust the ongoing movement. In Experiment 2 it jumped just before the finger tapped the screen. We anticipated that participants would respond to the resulting error by changing the way they moved on the next trial. The target diameter was adjusted to achieve 50% successful trials for both target speeds. In the control Experiment the target size was fixed and the target had to be intercepted within a green, square interception zone (centred 5 cm to the right of the centre of the starting point). The background was white rather than grey to make the interception zone easier to see. Further details are given in Table 1

The experiments were conducted in a normally illuminated room. Images were back-projected at 120 Hz and a resolution of 800 × 600 pixels onto a large screen (Techplex 150; 1.25 m x 1.00 m) that was tilted backward by 30° (Fig. 2). The main difference between the trials within each experiment was the target’s speed. There were slow targets and fast targets. Details of the target speeds and the targets’ lateral starting positions, as well as other differences between the experiments are summarised in Table 1 and Fig. 3. In the next paragraphs, we will explain the origin of these differences. Each experiment consisted of two or three blocks of trials.

In Experiment 1 we considered that participants might move slowly to have more time to adjust the ongoing movement. Therefore, we let the targets appear quite far to the left of the starting point. To make this possible, we placed the starting point 40 cm to the right of the screen centre. Since participants did not move slowly in Experiment 1, we placed the starting point only 20 cm to the right of the screen centre in Experiment 2. The starting point was larger in Experiments 2 than in Experiment 1 (Fig. 3). The reason for having a small starting point in Experiment 1 is that in that experiment the finger leaving the starting point initiated the target jump, so a smaller starting point ensures a more consistent timing across trials. In Experiment 2 the size of the starting point is irrelevant, so we selected a size that made it easy for participants to initiate trials. In the control of Experiment 2, we indicated a region within which participants had to hit the target to evaluate whether participants can adjust the timing of their movements more when they are discouraged to change *where* they hit the target.

The lateral position at which the target appeared was attuned to the target’s speed such that the target would be hit within about the same region of the screen when moving fast as when moving slowly. The change in the starting point’s colour between Experiments 1 and 2 was unintentional, and is presumably irrelevant. The target size intentionally changed in Experiment 2. It was larger in the control of Experiments 2 than in Experiment 1 to ensure that participants hit an acceptable number of targets (and so did not get too frustrated by target jumps that they could not compensate for). In the main part of Experiment 2 we equated performance rather than target size between slow and fast targets. To achieve this, targets initially had a diameter of 2 cm, but the diameter was multiplied by 1.1 every time the participant missed a target, and it was divided by 1.1 every time the participant hit the target. This was done separately for slow and fast targets (that were presented in separate blocks). Thus, on average, participants hit about half the targets. As it is more difficult to hit faster targets, we anticipated that fast targets would on average be larger than small ones. We increased the difference in speed between slow and fast targets after Experiment 1, because in Experiment 2 we expected to see changes to the next movement rather than to the current one, and adjustments to the next movement are usually incomplete (van Beers 2009).

### Experimental setup and measurements

An infrared camera system (Optotrak 3020, Northern Digital, Waterloo, Ontario) was placed at about shoulder height to the left of the screen (Fig. 2). It measured the position of a marker (an infrared light emitting diode) attached to the nail of the participants’ right index finger at 500 Hz. At the beginning of each block of each experiment, participants aligned the Optotrak’s coordinate system with the screen by placing their right index finger on four small dots at the corners of an imaginary 60 cm x 50 cm rectangle at the centre of the screen. The marker’s positions when the finger was on the four dots were used to express later finger movements with respect to the screen, automatically correcting for the fact that the marker was attached to the fingernail rather than to the tip of the finger.

To know where the finger is with respect to the moving target, we also had to synchronize the measured marker positions with the presentation of the images of the moving target. For this, we presented a flash at the top-left corner of the screen at the moment a new target appeared. A similar flash was presented at the moment the target jumped. About 1 ms after the flash stimulated a sensor that was placed in the path of the light projected towards the top-left corner of the screen, a second marker attached to the left side of the screen stopped emitting infrared light for about 10 ms. This second marker ‘disappearing’ in the Optotrak measurements was used to synchronize the timing of the finger movements with the images. It did so to within 2 ms.

A tap on the screen was detected when the finger was less than 0.5 cm above the screen, and its deceleration in the direction of the screen (or acceleration away from the screen) was larger than either 50 m/s^2^ (Experiment 1) or 40 m/s^2^ (Experiment 2). We reduced the threshold after Experiment 1 because sometimes participants tapped too gently. When they do so we can recover the moment of the tap during the analysis, but the participant does not receive the appropriate feedback. In Experiment 1 this is not really a problem, but in Experiment 2 seeing the tapping error is obviously critical.

Once a tap was detected, we determined whether the target was hit by comparing the position of the finger at the moment of the tap with the position of the target at that moment. If the finger hit the screen before the target jump was visible on the screen (because the last 5 cm of the finger’s movement was covered within the delay) the feedback was determined using what the target’s position would have been if it had already jumped. A target was considered to have been hit if the participant’s fingertip (as determined from the position of the marker) was within the outline of the target at the moment of the tap. If so, the participant heard a sound and the target stopped on the screen at the position at which it was hit (due to the delay it was actually presented at several positions along its original path after being hit, but then jumped back to precisely where it had been at the moment of the tap; participants did not notice this). If participants missed the target, no acoustic feedback was provided and the target deflected away from the finger at 1 m/s (so if participants tapped below and to the right of the target, the disk moved up and to the left from where it had been at the moment of the tap). The static (if the target was hit), deflected (if it was missed), or continuing (if no tap was detected) target disappeared after 500 ms if it had not moved off the screen before then. In the control of Experiment 2, the sound indicating that the target had been hit successfully only sounded if the tap was within the indicated hitting region. If the target was hit outside this region it did stop moving, but there was no sound.

**Table 1:** Blocks, trials and target details

Experiment	1	2	2 control
**Blocks**	Slow, Fast, Interleaved	Slow, Fast	Slow, Fast
**Total number of trials per participant**	320	400	400
**Slow target speed (cm/s)**	40	25	25
**Fast target speed (cm/s)**	100	110	110
**Slow target appears left of starting point (cm)**	40	15	15
**Fast target appears left of starting point (cm)**	80	66	60

### Procedure

At a random moment between 0.6 and 1.2 s after participants placed their finger on the starting point, the starting point disappeared and the target disk appeared. If the finger left the starting point before the target appeared, the target did not appear, and the finger had to move back to the starting point to restart the waiting period. In half the trials the target jumped to the left and in the other half it jumped to the right. The idea was to compare participants’ fingers movements after leftward and rightward target jumps to identify how movements changed in response to seeing the target at a different position than anticipated. By doing so for targets moving at two different velocities, we aimed to determine whether participants rely more on changing the timing (*when* they tapped) than the position (*where* on the screen they tapped) when the target moved faster. The target jumps were always much smaller than the diameter of the target (Fig. 3). We know that people respond to even smaller jumps (Brenner et al. 2023). The target jumped as often to the left as to the right for each target speed, and the leftward and rightward jumps were always randomly interleaved. In Experiment 1 we presented slow and fast targets in separate blocks of 80 trials each, as well as randomly interleaved in a block of 160 trials (Table 1). In Experiment 2, we only presented slow and fast targets in separate blocks, because we expected the clearest influence on the next trial when the target was moving in the same way. In all cases, the order of the blocks was counterbalanced across participants. Participants could rest for a few minutes between the blocks.

### Data analysis

We determined the fraction of targets that were hit for each speed in each experiment, but other than that we made no distinction between hits and misses. Thus, we determined the time taken on each trial as the time from when the target appeared until the finger hit the screen, irrespective of whether or not the target was hit. Our main measure was the extent to which people adjusted where (position) and when (timing) they hit the screen. By comparing adjustments to leftward and rightward target jumps, we isolated responses to the ‘errors’ introduced by such jumps from other aspects of the movement. Experiment 1 examines changes to the ongoing movement, so we compared movements in which the target jumped leftward and rightward. Experiment 2 examines changes on subsequent movements, so we compared movements in which the target had jumped leftward and rightward during the previous trial. In both cases there is reason to believe that participants will change either the position, or the timing, or both (Brenner et al., 2015, 2023). Our prediction was that people would adjust the timing more (and position less) for faster targets.

Since the time (*t*) and lateral position (*x*) of the tap may gradually shift during the experiment as participants become more accustomed to the task, get tired, or learn from feedback on previous trials, we quantified the responses to the jumps that we introduced as the change relative the previous trial, rather than as changes relative to average behaviour. So, for each trial *n*, we calculated the signed trial-to-trial change in the lateral position of the tap on the screen ($\:\varDelta\:{x}_{n}$) and in the time taken ($\:\varDelta\:{t}_{n}$):

1 $$\:\varDelta\:{x}_{n}={x}_{n}-{x}_{n-1}$$

2 $$\:\varDelta\:{t}_{n}={t}_{n}-{t}_{n-1}$$

we related these changes to the direction of the jump on the current trial in Experiment 1, and to that on the previous trial in Experiment 2. Thus, if the target jumped to the left on trial *n* of Experiment 1, the values of $\:\varDelta\:{x}_{n}$ and $\:\varDelta\:{t}_{n}$ were considered to be responses to leftward jumps. If it jumped to the right, they were considered to be responses to rightward jumps. Similarly, if the target jumped to the left on trial *n*−1 of Experiment 2, the values of $\:\varDelta\:{x}_{n}$ and $\:\varDelta\:{t}_{n}$ were considered to be responses to leftward jumps. If it jumped to the right, they were considered to be responses to rightward jumps. In both cases, we then determined the median value for leftward and rightward jumps for each participant. We did so separately for blocked and interleaved trials in Experiment 1. We determined the median rather than the mean so that we do not need to worry about outliers. Our estimate of participants’ adjustments is half the difference between their median changes after rightward and leftward jumps. We divided these median changes by 1 cm (for the change in position) or the time it took the target to move 1 cm (for the change in timing) to express the changes in position and timing as fractions of the adjustment that is required to fully compensate for the target jump.

We predict that participants will adjust the timing more and the position less for fast targets than for slow targets. To test this prediction, we compared the difference between the changes in timing and position (expressed as fractions of the required adjustments) for slow and fast targets. We tested the hypotheses that this difference would be larger (more positive) for fast targets, indicating that participants rely more on adjusting the timing for fast targets, using paired one-sided t-tests. To get more insight in the way movements are adjusted, we also plot the means of the median changes (across participants), both as fractions of the values needed to compensate for the jump, and in terms of the actual changes (in mm for position and ms for timing). Since full compensation could be achieved by many combinations of adjustments to the timing and to the position of the tap, we plot the mean values of both adjustments with 95% confidence ellipses (across participants’ median values) for the combined means. Doing so can inform us on the extent to which differences between participants in the extent to which the time or position of the tap are adjusted are due to differences in the overall amount of compensation, or to differences in the way in which the compensation is achieved.

## Results

Participants hit a larger fraction of slow targets than of fast ones (Table 2). This was to be expected. That the effect of target speed on performance was much smaller for Experiment 2 is not surprising, because we tried to counteract differences in performance by adjusting the target’s size such that participants hit about half the targets in each block in that experiment. On average, this resulted in a target radius of 1.6 cm for fast targets and 1.1 cm for slow targets. Irrespective of the differences in performance and target size, participants took more time to hit slow targets (Table 2). This too was to be expected (Brouwer et al. 2000), but the difference might have been reinforced by the choice of the target’s starting positions here, especially in the control of Experiment 2 where participants had to hit the target within a certain region.

**Table 2:** Percentage of targets hit and median time taken to tap (mean ± standard deviation across participants for each target speed in each experiment). Values are for speeds presented in separate blocks, with values for interleaved speeds in experiment 1 within brackets

Experiment	1		2		2 control	
Target speed	Slow	Fast	Slow	Fast	Slow	Fast
Targets hit (%)	93 ± 6 (92 ± 6)	67 ± 13 (65 ± 13)	50 ± 1	49 ± 1	94 ± 6	59 ± 16
Time taken (ms)	702 ± 100 (692 ± 88)	666 ± 68 (684 ± 56)	707 ± 83	610 ± 42	740 ± 36	591 ± 8

**Fig. 4:**
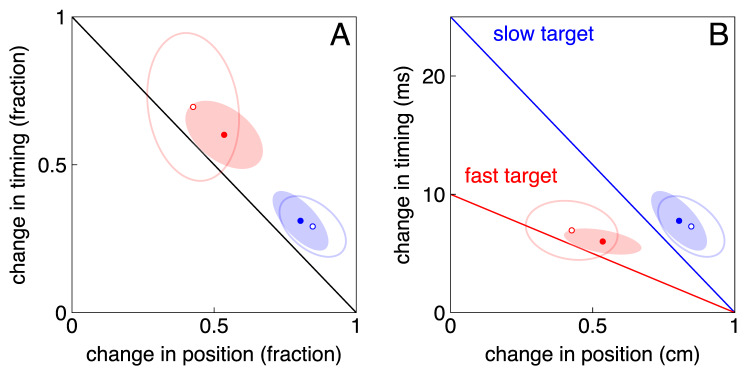
Results of Experiment 1: adjusting ongoing movements. **(A)** Fraction of the jump that is compensated. **(B)** The compensation in metric units. Lines: options for full compensation for the target jump. Dots: actual mean compensation with 95% confidence ellipses. Filled symbols and ellipses: slow and fast targets presented in separate blocks. Open symbols and ellipses: slow and fast targets randomly interleaved within a single block

On average, in Experiment 1, participants had 345 ms to adjust the ongoing slow movements and 308 ms to adjust the ongoing fast movements when the speeds were presented in separate blocks. They had 351 ms for slow movements and 314 ms for fast movements when the speeds were randomly interleaved. This is the time between the target jumping and the finger tapping on the screen. Within this time, they fully compensated for the target jumps (Fig. 4). On average, they even slightly over-compensated (points slightly above the lines; three of the four 95% confidence intervals do not even intersect the line). In line with our hypothesis, the fraction of the jump that was accounted for by changing the timing was larger for faster targets (red symbols are further to the top left than their blue counterparts in Fig. 4A), both when slow and fast targets were presented in separate blocks (t_43_ = 4.31, *p* < 0.0001), and when they were randomly interleaved (t_43_ = 4.19, *p* < 0.0001).

A closer look at the changes in timing and position when the target jumps (Fig. 4B) reveals that participants did not adjust their timing (in ms) more for fast targets than for slow ones, but they did adjust the position (in cm) less. So, the relative contributions of changes in timing and position were adjusted in the way we predicted, but the actual change in timing did not depend on the target’s speed; only the change in position did. The elongation of the ellipses along the lines of full compensation suggests that some of the variability across participants is the result of different participants adjusting timing and position to different extents to achieve the same overall magnitude of adjustment.

**Fig. 5:**
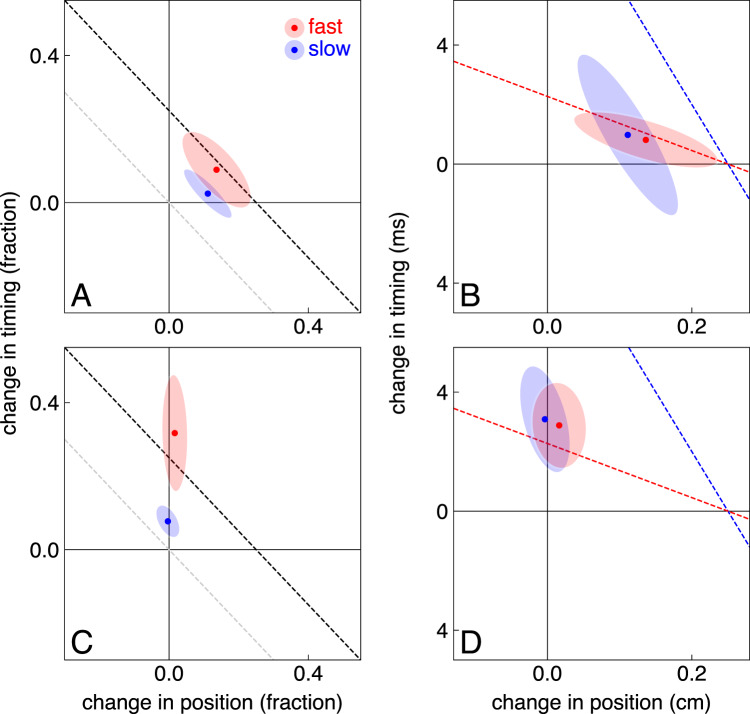
Changes to the next movements in Experiment 2. Same symbol and colour use as in Fig. 4, but the scaling of the axes is different. The dashed lines represent 25% compensation for the error introduced by the jump on the previous trial, except for the grey lines in **A** and **C** that represent changes that provide no net compensation. **A** and **B** show data for the main experiment. **C** and **D** show data for the control experiment in which an interception region was indicated

As expected, participants only partially compensated for the errors introduced by the target jumps in Experiment 2, so we plot lines for 25% compensation rather than full compensation in Fig. 5. In the main part of Experiment 2, participants compensated for almost 25% of the error for fast targets, but less for slow targets (Fig. 5A). In contrast to our hypothesis, they did not rely significantly more strongly on adjusting the timing for fast targets than for slow targets (t_23_ = 0.35, *p* = 0.37). The elongation of the ellipses along the lines shows that there is more variability across participants in the distribution of the change between timing and position, than in the combined magnitude of the change. Looking at the metric changes (Fig. 5B), we see that on average participants changed both the timing and the position on the next trial by about the same amount for slow and fast targets: about 1 ms and 1 mm, respectively.

We can be confident that participants adjusted their movements in response to the target jumps in Experiment 2, because the 95% confidence ellipses do not intersect the dashed grey line in Fig. 5A. We can also be confident that they adjust the position of the tap (confidence intervals do not intersect the vertical axis in Fig. 5B). But we cannot be confident that participants adjust the timing of the tap (confidence intervals do intersect the horizontal axis in Fig. 5B). We therefore conducted a control experiment in which we encouraged participants to adjust the timing by indicating approximately where they should hit the target. This stopped participants from adjusting the position (horizontal values close to zero in Fig. 5C and D). And indeed, they now clearly adjusted the timing of the tap (confidence intervals do not intersect the horizontal axis). The fraction of the target jump that was compensated for by changing the timing of the next tap was significantly larger for fast targets (t_16_ = 2.16, *p* = 0.023; confidence intervals do not overlap in Fig. 5C), but again this dependence on target speed was realised without adjusting the actual change in timing. The change was about 3 ms, irrespective of the target speed. It was larger than the 1 ms that we found in the main experiment, presumably because only the timing can freely be adjusted, implying that the metric extent of the change in timing can be modified. It just does not appear to be modified in relation to the target speed.

## Discussion

We confirmed that participants can adjust both the position and the timing of their attempts to tap on moving targets by showing how the movements change in response to target jumps. Such changes take place during the movement if there is enough time. If not, the target jumps give rise to errors, a fraction of which is compensated for in the next movement. Our question was whether participants would rely more on adjusting the timing if the target was moving faster. The fraction of the target jump that was compensated for by changing the timing was indeed sometimes larger for fast targets than for slow ones (Figs. 4A and 5C), but it was clear that the metric change in timing did not increase with target speed (Figs. 4B and 5B and D). The same metric change in timing compensates for more of the 1 cm target jump if the target is moving faster. In Experiment 1, in which the compensation is complete, a larger part of the compensation is achieved by the change in timing. And the position changes less. In Experiment 2 the compensation is incomplete, and the change in timing seems to mainly increase the overall amount of compensation (Fig. 5A). Importantly, even in Experiment 1 and in the control for Experiment 2, where adjusting the timing accounts for a significantly larger fraction of the correction for fast targets, this is clearly the consequence of the target moving further during the adjusted time period, rather than of the timing being adjusted more.

### Corrections to ongoing movements (Experiment 1)

The pattern of changes that we see in Fig. 4 is consistent with our earlier claim that the timing of the movement is adjusted first, and the vigour of positional adjustments are tailored to the target’s speed to complete the correction within the remaining time (Brenner et al. 2022). In Experiment 1, the change in timing was about 7 ms, irrespective of the target’s speed. This change contributed more to the required adjustment when the target moved faster, because the target moved 2.5 times further within that time. Consequently, for faster targets, the position did not need to be adjusted as much to achieve the same overall level of adjustment. Thus, although participants did not change the timing of their movement more for fast targets, a larger fraction of the correction was achieved by changing the timing. We found no clear difference between blocking and interleaving the target speeds.

### Adjusting the next movement (Experiment 2)

If there is an error at the moment of the tap, one can use this error to adjust the next similar movement. In Experiment 2, the next target moved as similarly as possible on all trials of a block. The only difference between trials was that we randomly interleaved leftward and rightward jumps. If we had not varied the direction of the jump, the error would probably quickly have disappeared as participants adjusted their movements in the direction of the jump: ending up tapping later and further to the left if the target repeatedly jumped to the left, and sooner and further to the right if the target repeatedly jumped to the right (Brenner et al. 2016). The adjustments to the next movement were weaker here than in an earlier study in which the tapping errors arose from ignoring acceleration (Brenner et al. 2023). In Experiment 5 of that study, we inferred that a fraction of about 0.25 was corrected on the next trial by changing the position, and a fraction of about 0.15 by changing the timing. That would correspond with changes of 2.5 mm, and changes of 6 ms for slow targets and 1.4 ms for fast targets, in the current study. The average target speed in the previous study was between the two speeds of the current study, so both the changes (about 1 ms and 1 mm) are smaller in the current study. In studies with static targets, the reported (spatial) correction per trial is about 0.4 (van Beers 2009), or even slightly more (van der Kooij et al. 2015). Here, the overall correction is less than 25% for fast targets, and even less for slow targets (Fig. 5C). In the control of Experiment 2 the correction is more than 25% for fast targets, but still less than 40%. It is even less than in Experiment 2 for slow target. We have no idea why the corrections were so much smaller in Experiment 2 of the present study.

## Conclusion

We find no evidence that people adjust their timing more for fast targets than for slow ones; neither in on-line adjustments nor in trial-to-trial adjustments. They do not need to, because any change that they make to the timing has more impact if the target is moving faster. If there is enough time to adjust the ongoing movement, the timing is adjusted modestly, irrespective of the target speed. The rest of the adjustment is done by changing the position at which the target is hit, and thus the finger’s trajectory. Consequently, the fraction of the adjustment that is achieved by the fixed change in timing is larger if the target is moving faster. If there is no time to adjust the ongoing movement, fixed temporal and spatial corrections appear to be applied on the next attempt, irrespective of the target’s speed.

## Data Availability

Data is provided at https://osf.io/tjxg3/.
